# The Prevalence of Multidrug-Resistant *Escherichia coli* Producing ESBL among Male and Female Patients with Urinary Tract Infections in Riyadh Region, Saudi Arabia

**DOI:** 10.3390/healthcare10091778

**Published:** 2022-09-15

**Authors:** Adil Abalkhail, Ahmad S. AlYami, Saeed F. Alrashedi, Khalid M. Almushayqih, Thamer Alslamah, Yasir Ahmed Alsalamah, Ayman Elbehiry

**Affiliations:** 1 Department of Public Health, College of Public Health and Health Informatics, Qassim University, Al Bukayriyah 52741, Saudi Arabia; 2 King Fahad Medical City, P.O. Box 59046, Riyadh 11525, Saudi Arabia; 3 Ministry of Health Qassim Region, Buraydah 52367, Saudi Arabia; 4 General Surgery Department, Unaizah College of Medicine, Qassim University, Unayzah 56453, Saudi Arabia

**Keywords:** antimicrobial resistance, ESBL-*E. coli*, urinary tract infection

## Abstract

The *Escherichia coli* that produces extended-spectrum lactamases (ESBL-*E. coli*) can develop resistance to many antibiotics. The control of ESBL-*E. coli* disorders is challenging due to their restricted therapeutic approaches, so this study aims to determine the prevalence and pattern of the antibiotic resistance of ESBL-*E. coli* among male and female patients with urinary tract infections in Riyadh, Saudi Arabia. During the period of 2019 to 2020 at King Fahd Medical City, Riyadh, 2250 urine samples from patients with urinary tract infections (UTIs) were collected, and microbial species were cultured and identified using standard biochemical techniques. A double-disc synergy test was used to identify ESBL-producing strains of *E. coli,* and an in vitro method and the clinical laboratory standard institute (CLSI) criteria were employed to determine the resistance of these strains to antimicrobial drugs. ESBL-*E. coli* was detected in 510 (33.49%) of the 1523 *E. coli* isolates, 67.27% of which were recovered from women and 33.7% of which were recovered from men. A total of 284 (55.69%) ESBL-*E. coli* isolates were found in patients under 50 years of age, and 226 (44.31%) were found in patients over 50 years of age. Nearly all the isolates of ESBL-*E. coli* were resistant to cephalosporins (ceftriaxone, cefotaxime, cefepime, cefuroxime, and cephalothin) and penicillin (ampicillin), whereas the majority of the isolates were sensitive to several carbapenems (imipenem, meropenem, and ertapenem), aminoglycosides (amikacin), and nitrofurantoins. The development of antibiotic resistance by ESBL-*E. coli*, the most frequent pathogen linked to urinary tract infections, plays a crucial role in determining which antibiotic therapy is appropriate.

## 1. Introduction

Urinary tract infection (UTI) is a broad term that describes a bacterial infection or inflammation of any region of the urinary system [1]. One of the most predominant bacterial disorders encountered by doctors globally is a UTI [2,3]. Furthermore, because antibiotic sensitivity results in reported cases of UTI must be recorded within 48 h after sampling, the illness is handled therapeutically depending on the available antibiotic resistance data [4]. A UTI can be categorised into two types: a complex infection or a simple infection. The term “complex” refers to a patient’s urinary tract being obstructed or aberrant in some way. Simple infections are significantly more prevalent and refer to infections that occur within a normal, unobstructed urinary tract [5]. If a urinary tract infection (UTI) is not discovered and treated early enough, it can lead to chronic sickness and long-term kidney impairment [6]. Patients with uncomplicated UTIs can be treated because the causal bacteria are relatively predictable: *E. coli* causes 80–90% of UTIs [7].

In clinical practice, UTIs are the most prevalent bacterial infection, affecting people of all ages, particularly women [8,9]. Gram-negative bacteria cause 80–85% of UTIs, and *Escherichia coli* (50–70%) is the most common causal organism, followed by *Klebsiella* [10,11]. As a member of the *Enterobacteriaceae* family, *E. coli* is the most prevalent causal bacteria linked with urinary tract infections, accounting for up to 80% of all cases [12,13]. In the past 20 years, antibiotics have been widely used to treat pathogenic bacteria; however, the overuse of these drugs has resulted in the establishment of antimicrobial resistance around the world, a concern that has become a serious global health risk in recent years [14,15].

With increasing antimicrobial resistance and their potential influence on patient safety, multidrug-resistant organisms and their impacts on surveillance systems are both a concern [16,17]. There is no doubt that the emergence of bacteria resistant to antibiotics is a serious public health concern [18,19]. A shortage of novel antibiotics makes the problem of treating many illnesses even worse due to the rise in resistance across many nations. Antimicrobial resistance (AMR) infections kill approximately 700,000 individuals globally each year, with the figure expected to rise to 10 million by 2050 [20]. Urinary tract infections (UTIs) are the most predominant infection, ranging from basic cystitis to urosepsis and septic shock [21,22]. The ESBL prevalence rates between studies may be influenced by geographical differences. Incidences of antibiotic resistance or ESBLs may also be overestimated or underestimated depending on the inclusion and exclusion criteria of isolates [23].

B-lactam inhibitors are the safest and perhaps habitually given antibiotics for urinary tract infections [3]. B-lactam inhibitors, according to Ahmed et al. [24] and Rajabnia et al. [25], are a form of broad-spectrum antibiotics containing a beta-lactam ring in their composition. Numerous findings demonstrate that *E. coli* in UTIs is more resistant to beta-lactamase drugs [26,27]. An additional source of concern is the development of ESBL-generating microorganisms, which lyse the beta-lactamase ring, rendering the antibiotic ineffective and slowly decreasing the number of viable therapy options [25,28].

The primary microorganism-encoding-developed ESBL was discovered in Germany in 1983, and ESBL-*E. coli* has spread globally in both public and medical centres since 2000 [25,29]. Concerning this, the prevalence of ESBL-*E. coli* continues to rise worldwide [13,30]. A prolonged hospital length of stay, a significant rise in antibiotic usage, and, as a result, higher hospital expenses, as well as a trend towards higher death rates are all outcomes of this scenario [31,32,33]. Beta-lactam inhibitors, particularly carbapenems, have long been thought to be the best treatment for ESBL-*E. coli* outbreaks [34,35,36]. The increase in beta-lactam-resistant bacterial pathogens around the world, on the other hand, poses a danger to this antibacterial family’s efficacy [37,38]. Moreover, sulfonamide and fluoroquinolone resistance is common, reducing the possibility of alternative therapeutic options [32,39].

It is critical to examine the antimicrobial resistance patterns of *E. coli* in actual community-acquired illnesses in order to comprehend the level of resistance and to select the most effective empirical antibiotic therapy for UTIs [23]. Because ESBL-*E. coli* and its antimicrobial sensitivity profiles have received poor attention in Saudi Arabia [40,41], this type of research would therefore assist physicians to choose a suitable empirical antimicrobial therapy. As a result, the purpose of this research was to identify and assess ESBL-*E. coli* antibiotic resistance in patients with urinary tract infections in King Fahd Medical City in Riyadh, Saudi Arabia.

## 2. Materials and Methods

### 2.1. Design of the Study and Data Collection

From 2019 to 2020, the King Fahd Medical City (KFMC) Microbiology Laboratory conducted a randomised experimental study (nonprobability sampling) of all ESBL-*E. coli* strains detected in urine samples. Patient data were collected using the clinical microbiology laboratory service management system. The study included patients who had a clinically suspected UTI. We included all individuals whose urine cultures were positive for *E. coli* in this study. Urine specimens for which a particular microbial species was cultivated at a level of more than 10^5^ CFU/mL were characterised as showing substantial monomicrobic bacteriuria (urine bag). The urine samples of individuals with multimicrobic bacteriuria or those collected more than three days after being admitted to the hospital were discarded.

To prevent the overestimation of resistance rates from various urine samples, only the initial isolate of *E. coli* from each individual was used in this investigation, which included 2250 urine samples. Each isolate was divided into two categories: outpatient and inpatient. Outpatient samples came from the hospital’s outpatient departments as well as from outside the hospital from wide-ranging physicians in the neighbourhood. Medical samples were obtained from both the emergency unit and several inpatient units. The obtained samples were correctly registered and transferred to the microbiological laboratory for further analysis in accordance with current European guidelines. The specimens were kept chilled at 4 to 6 °C when rapid transportation was not available [42].

### 2.2. Culturing of Samples

Cultures were taken from all of the urine samples using a semiquantitative technique provided in the World Health Organization (WHO) guidelines on regular culture media [43]. In brief, 1 mL of urine was streaked over cystine-lactose-electrolyte-deficient (CLED) and blood agar plates (Hardy Diagnostics, Santa Maria, Santa Maria, CA, USA) and incubated aerobically for 24 h at 37 °C using a calibrated wire loop. Biochemical tests and cultural traits were used to describe and identify the isolates using the BD Phoenix (BD – Canada, Mississauga, ON, Canada) and API ID (bioMerieux UK Ltd, Basingstoke, UK) systems.

### 2.3. Identification of E. coli Isolates Using the BD Phoenix System

The NMIC/ID-4 panel of the Phoenix system was used to examine a total of 1523 G-negative bacteria. First, the McFarland 0.5 stock solution was prepared in 4.5 mL of Phoenix ID broth [44] using a Sensititre™ Nephelometer (Thermo Fisher Scientific, Waltham, MA, USA). The obtained bacterial ID solution was placed in the ID field of the Phoenix panel. On the panel, each chemical reactivity well was soaked with 50 µL of microbial solution. Any extra suspension was collected by the absorbing cushion at the underside of the panels. After being sealed with a rubberised coating and reading the display code, the panel was placed directly into the Phoenix machine. The Phoenix system ID results for 1523 cultivated *E. coli* isolates were matched with those of the traditional API system as standards. After that, accurate ID rates were determined at the genus and species levels.

### 2.4. Determination of the Antimicrobial Susceptibility of E. coli Strains Using the Kirby Bauer Method

Depending on the Clinical Laboratory Standard Institute (CLSI) recommendations version 6.0, all detected *E. coli* isolates were investigated in vitro for antimicrobial sensitivity through the agar disc diffusion technique (Hardy Diagnostics, Santa Maria, Santa Maria, CA, USA) [45]. The following antibiotics were tested in our research: ampicillin (10 μg), amoxicillin-clavulanate (20/10 μg), cephalothin (30 μg), cefuroxime (30 μg), ceftazidime (30 μg), cefoxitin (30 μg), cefepime (30 μg), cefotaxime (30 μg), ceftriaxone (30 μg), ciprofloxacin (5 μg), gentamicin (10 μg), amikacin (30 μg), trimethoprim-sulfamethoxazole (1.25/23.75 μg), piperacillin-tazobactam (36 μg), imipenem (10 μg), meropenem (10 μg), ertapenem (10 μg), levofloxacin (5 μg), tigecycline (15 μg), nitrofurantoin (300 μg), colistin (10 μg), and aztreonam (30 μg). The experimental analysis (S/I/R) of the findings takes into account the CLSI’s suggested diameters or breakpoints [45]. If an isolate proved resistant to three different antibiotic groups, it was classified as multidrug resistant.

### 2.5. Screening of ESBL-E. coli with a Disc Susceptibility Test

Three markers for cephalosporins, including ceftazidime (30 g), cefotaxime (30 g), and cefpodoxime (30 g), were used to evaluate all strains for ESBL-*E. coli*. If the zone diameter for the three markers was 22, 27, or 17 mm, the isolates were termed resistant. The isolates that demonstrated tolerance to at least one of the three cephalosporins were subjected to phenotypic confirmatory tests [46,47].

### 2.6. Double-Disc Diffusion Test (DDST)

The DDST-evaluated *E. coli* strains that were resistant to each of the three antibiotics used for ESBL development. ESBL detection was performed using ceftazidime (30 g), cefotaxime (30 g), cefpodoxime (30 g), and amoxicillin/clavulanic acid (amoxicillin 20 g + clavulanic acid 10 g) [48]. On Muller–Hinton agar (MHA) plates, amoxicillin/clavulanic acid (20 g/10 g) and the discs of third-generation cephalosporins were placed at a distance of approximately 20 mm from centre to centre. The plates were incubated at 37 °C overnight. Any increase in the cephalosporin inhibition zone in the direction of the amoxicillin/clavulanic acid disc was observed as a confident ESBL finding. As controls, ATCC 700603 *Klebsiella pneumoniae* and ATCC 25922 *E. coli* were employed.

### 2.7. Calculation of the Multiple Antibiotic Resistance (MAR) Index

The MAR index is a tool for assessing potential hazards and checking antibiotic-resistant bacteria. There were 22 antimicrobial agents included in this trial, which are represented as (b). Based on the method outlined by Osundiya et al. (2013), the MAR index was calculated by dividing the number of antimicrobial drugs to which the isolate conferred resistance (a) by the number of antimicrobial drugs utilised throughout the investigation (b) [49].

### 2.8. Statistical Analysis

Our data were analysed using SPSS Software 21.0 (SPSS, Chicago, IL, USA).

## 3. Results

Throughout the course of the study, 2250 urine specimens that fulfilled the inclusion criteria were taken from individuals with urinary tract infections in various departments at KFMC. A total of 1523 specimens (67.68%) containing *E. coli* isolates were obtained from individuals with nonrepetitive UTIs, with 510 (33.49%) containing ESBL-*E. coli* and 1013 (66.51%) containing non-ESBL-*E. coli*. The percentages of female and male patients with ESBL-*E. coli* were 66.27 and 33.73%, respectively, while the percentages of female and male patients with non-ESBL-*E. coli* were 79.57 and 20.43%. According to our findings (Table 1), the *E. coli* spectrum in male and female urinary tract infection patients differs significantly, and females were significantly more likely to acquire the infection than males.

As shown in Table 1, the proportions of the non-ESBL-*E. coli* and ESBL-*E. coli* samples among female and male patients over 50 years of age were 622/1523 (48.84%). The proportions of the non-ESBL-*E. coli* and ESBL-*E. coli* samples among female and male patients 50 years of age or younger were 901/1523 (59.16%). Based on the age-related spectrums of *E. coli*, the results showed that ESBL-*E. coli*- and non-ESBL-*E. coli*-related UTIs were predominant in age groups less than 50 years old, with a higher proportion of 415/901 (46.1%) being seen in the age group between 31–40 years old.

Based on the information presented in Table 2, we used seven classes of antibiotics, including four subclasses of beta-lactam antibiotics: cephalosporins (ceftriaxone, cefotaxime, ceftazidime, cefepime, cefuroxime, cephalothin, and cefoxitin), penicillins (ampicillin, amoxicillin-clavulanate, and piperacillin-tazobactam), carbapenems (imipenem, meropenem, and ertapenem), and monobactams (aztreonam). The other classes were sulfonamides (trimethoprim-sulfamethoxazole), aminoglycosides (gentamicin and amikacin), tetracycline (tigecycline), fluoroquinolones (ciprofloxacin and levofloxacin), nitrofurantoin, and polymyxin (colistin). All the ESBL-*E. coli* strains tested in the study were resistant to ampicillin (100%) as a member of the penicillin subclass and all cephalosporins except cefoxitin had more than or equal to 97% resistance. Other tested antibiotics, such as amoxicillin-clavulanate, tigecycline, and trimethoprim-Sulfamethoxazole, also proved resistant to between 60 and 90% of all ESBL-producing *E. coli*. On the other hand, the ESBL-*E.* *coli* strains were more sensitive to carbapenems (ertapenem, imipenem, and meropenem) and aminoglycosides (amikacin). As a result of our study, we found an increase in the antimicrobial resistance among *E. coli* strains towards the use of conventional antibiotics, which indicates the need for surveillance programs aimed at monitoring antimicrobial resistance.

Multidrug-resistant strains of ESBL-*E coli* were investigated, and the majority of those screened showed resistance to three or more of the tested antibiotics. Table 3 illustrates the multiple antibiotic resistance (MAR) index of the 510 ESBL-*E. coli* strains in the UTIs. The average MAR index of all the ESBL-*E. coli* strains was 0.36. A total of 163 out of 510 (31.96%) ESBL-*E. coli* strains revealed resistance against 15 antimicrobial drugs, including CRO, CTX, CAZ, FEP, CXM, USAN, AMP, AMC, ATM, SXT, TGC, CIP, LVX, CST, and TZP, with a high MAR index of 0.68, whereas only 4.31% of the isolates were resistant to 5 antimicrobials (CRO, FEP, CXM, AMC, and LVX). The MAR index for several strains was more than 0.2. The MAR index, on the other hand, for only 30 isolates (5.88%) was less than 0.2. As a result, the majority of the ESBL-*E. coli* bacteria were extremely resistant to numerous antimicrobial medicines that have been evaluated and had high MAR index values.

## 4. Discussion

The development and increasing prevalence of multidrug-resistant Enterobacteriaceae, particularly Enterobacteriaceae that produce ESBLs, concerns the entire globe. ESBL-*E. coli* has been recognised as a major pathogen, with both nosocomial and public outbreaks worldwide since the 2000s, the severity of which differs by location [13,50]. Knowing the regional demography and how it has changed over the years is essential for selecting the most effective first-line antimicrobial treatment for each area. We are conscious of the great weight we bear in our community as a result of a variety of variables woven into the features of medical practice, legislation, and the health care system [50,51].

Furthermore, ESBL-*E. coli* is more prevalent among women than men, and in hospitalisations, it accounts for a large proportion of all hospital-acquired infections and thus seems to be very dangerous [52]. ESBL-*E. coli* presents a substantial danger to uropathogen therapy, as well as to the effectiveness of last-line medicines [53]. Upwards of one-third of the *E. coli* strains in our investigation (33.48%) were ESBL-*E. coli* strains. Several earlier investigations [20,54,55,56] have repeatedly revealed a significant rate of ESBL-*E. coli* strains. According to Hashemizadeh et al. [57], 41% of outpatients in Kerman, Iran, had ESBL-positive isolates from UTIs. Ali et al. [58] found a similarly high rate (40%) of ESBL producers in Pakistani uropathogenic *E. coli*. ESBL-producing *E. coli* may be more frequent in Iran and other developing nations because of the widespread use of -lactam medications and the lack of tight regulation on their use.

Out of 1523 *E. coli* strains recovered from urine samples, 510 (33.48%) produced ESBLs, whereas 1013 (66.52%) did not. Parallel results were previously reported regarding the occurrence of ESBL-*E. coli* isolates recovered from patients with UTIs, such as 37.11% [25] and 46.87% [59]. The antimicrobial prescription strategy; the overuse of wide-ranging medications, particularly penicillin and third-generation cephalosporins; geographic differences; and hospitalisations may all contribute to these variances in the prevalence of ESBL-*E. coli* throughout UTIs [60,61]. Additionally, we found no significant impact on the incidence of recovered ESBL-*E. coli*. Nonetheless, as previously noted by Rajabnia et al. [25], Fernando et al. [33], and Rohde et al. [62], a rise in the proportion of recovered ESBL-*E. coli* has been recorded globally.

Sex, age, and hospitalisation have all been cited as associated factors in the previous literature [63,64]. The number of reported UTIs was higher in women, 1144 (75.11%), than in men, 379 (24.89%), in the current investigation of positive *E. coli* cases. The incidence of diagnostic ESBL-*E. coli* cases was 338 (66.27%) in women and 172 (33.73%) in men. Because of the architecture of their sexual organs, women are more commonly affected by UTIs, that is, the compact size and proximity of the urethra to the rectum encourage the spread of ESBL-*E. coli* [50]. Thus, the sex of the patient is a potential risk factor for UTIs (*p* = 0.008) that should be considered. Furthermore, the largest number of ESBL strains, along with the frequency of ESBL-*E. coli* microorganisms (284/510), were detected in people 50 years old or younger (55.69%). Along with the antimicrobial sensitivity findings, the strongest antibiotic resistance among ESBL-*E. coli* was demonstrated with the penicillin subclass (100% for ampicillin and 89.41 for amoxicillin-clavulanate), the cephalosporin subclass (99.80% for cephalothin, 99.61% for cefotaxime and ceftriaxone individually, 99.41% for cefuroxime, 98.43% for ceftazidime, and 97.45% for cefepime), the monobactam subclass (99.22% for aztreonam), and the tetracycline class (77.05% for tigecycline).

Moreover, the ESBL-*E. coli* strains showed a certain degree of resistance to fluoroquinolones (68.03% for ciprofloxacin and 64.50% for levofloxacin), sulfonamides (63.13% for trimethoprim-sulfamethoxazole), and polymyxin (58.43% for colistin). The highest degree of sensitivity was observed with carbapenem (100% for ertapenem, 99.80% for imipenem, and 99.61% for each meropenem) and aminoglycosides (100 for amikacin and 72.55% for gentamycin).

Our results are parallel with those of certain former investigations [50,53,56]. In the Aljouf district of Saudi Arabia, Bandy and Tantry [65] observed a significant frequency of multidrug-resistant Enterobacteriaceae. In a separate investigation, 88.3% of *E. coli* strains exhibited a strong resistance to ampicillin [66]. Nonetheless, when compared to previous studies in Canada (11%) and the United States (16–18%) [67], these values are regarded as extremely high. Furthermore, among the *E. coli* strains recovered from our urine samples, 100% were resistant to ampicillin, while this value was 67.2% in four African and Asian investigations [68]. This disparity could be explained by the ease with which these antimicrobial agents can be purchased from a retail pharmacy in our nation, as opposed to the strict constraints in place in other regions of the world, and the widespread inappropriate antibiotic use for illnesses such as the common cold, some ear infections, and runny noses in our country. The development of resistance to beta-lactamase and cephalosporin groups that we detected indicated that these drugs may no longer be appropriate for symptomatic treatment. Carbapenems and aminoglycosides have certainly been indicated as suitable potential therapies for UTIs based on our findings. With a tolerance percentage of 0.12% for imipenem, 0.39% for meropenem, 0.00% for ertapenem, and 0.39% for amikacin against *E. coli*, this seems acceptable. The increasing widespread usage of this antimicrobial, on the other hand, may address doubts about rising resistance rates in the coming years.

It is difficult to estimate the cost of infections caused by multidrug-resistant pathogens because the financial burden differs from health system to health system [17,69,70]. In spite of the difficulties in quantifying costs, it is plausible to show that this phenomenon negatively impacts the economy due to longer hospital stays, higher care costs, increased laboratory and diagnostic tests, the need for additional interventions, higher prices for the application of prevention and mitigation programs, and any legal proceedings that may follow [17].

Due to the constant changes in health care, technological innovation, regulatory structures, and a need to maximise the resources provided by health systems, it is essential to understand the costs of infections caused by multidrug-resistant organisms so that targeted prevention and control programs can be implemented that are increasingly cost-effective [17]. The analysis of hospital infections’ social and economic impacts is becoming increasingly important when it comes to implementing prevention and surveillance systems. It is imperative to involve professionals from a variety of disciplines in the design and implementation of successful intervention methods in order to receive relevant contributions [17].

This research will have a major effect on how UTIs are treated in Saudi Arabia’s Riyadh district. Our investigation, nevertheless, has some limitations. For instance, the study population is restricted to a single province in Saudi Arabia. This research was conducted in the Saudi Arabian city of Riyadh, and the results may not represent antibiotic resistance patterns in other areas of the country. A wider population of patients with a particular medical problem from a specific region of Saudi Arabia could provide more knowledge. For ESBL UTIs, the time needed for urine testing and reporting frequently hinders the administration of the proper antibiotic. It can lead to tremendous morbidity, including kidney scarring, hypertension, and renal impairment in the worst-case scenario. It also delays diagnosis and treatment. In our investigation, no such pronounced effects were discernible. Additionally, the molecular identification of ESBL-producing *E. coli* strains and in-depth phylogenetic studies may be more beneficial in understanding the dispersion of these bacteria in our environment.

## 5. Conclusions

Antibiotic therapy for UTIs caused by ESBL-*E. coli* no longer seems to be indicated because it promotes the increasing prevalence of antibiotic-resistant bacteria. Additionally, improper prescription use is linked to the growth of multidrug-resistant (MDR) microorganisms. Thus, to avoid the development of MDR microorganisms such as ESBL-*E. coli*, doctors should ensure that suitable drugs are prescribed and used at the proper periods and at measured settings. The current study demonstrates that the therapeutic prospects of beta-lactam antibiotics pose a significant issue and a major obstacle for physicians. UTIs induced by ESBL-*E. coli* have become more common in recent years. As a result, the early detection of infections by this organism is critical for prompt treatment and reduced fatality in community and hospital settings. Furthermore, resistance to antibiotics, particularly resistance by ESBL-*E. coli*, should be regularly assessed and evaluated at various time intervals. These results further imply that earlier and accurate approaches for detecting ESBL-producing bacteria should be employed to prescribe relevant antibiotics. When analysing the results of this study, some limitations have been identified, such as the fact that the study was conducted in only one health centre in the Riyadh region, which may not demonstrate the cyclic, pathological, and cultural diversity of pathogenic organisms and their antimicrobial sensitivity profiles across the country. Because of the shortage of time in the laboratory, the genotyping of the ESBLs in *E. coli* strains could not be determined.

## Figures and Tables

**Table 1:** A look at key risk variables in urine *E. coli* isolates from 2019 to 2020.

Factor	Non-ESBL-*E. coli* Isolates (*N* = 1013)	ESBL-*E. coli* Isolates (*N* = 510)	Total (*N* = 1523)
**Sex**			
Female	806 (79.57%)	338 (66.27%)	1144 (75.11%)
Male	207 (20.43%)	172 (33.73%)	379 (24.89%)
**Age**			
0–10 years	98 (9.67%)	49 (9.61%)	147 (9.65%)
11–20 years	52 (5.13%)	21 (4.18%)	73 (4.79%)
21–30 years	90 (8.88%)	31 (6.07%)	121 (7.94%)
31–40 years	280 (27.64%)	135 (26.47%)	415 (27.25%)
41–50 years	97 (9.58%)	48 (9.41%)	145 (9.52%)
≤50 years	617 (60.91%)	284 (55.69%)	901 (59.16%)
˃50 years	396 (39.09%)	226 (44.31%)	622 (40.84%)

**Table 2:** Degree of resistance of 510 ESBL-*E. coli* strains recovered from the urine samples of patients suffering from urinary tract infections.

Antimicrobial Drug	Resistant	
	No.	%
**Beta-lactam antibiotics**		
**Subclass (cephalosporins)**		
Ceftriaxone (CRO)	508	99.61
Cefotaxime (CTX)	508	99.61
Ceftazidime (CAZ)	502	98.43
Cefepime (FEP)	497	97.45
Cefuroxime (CXM)	507	99.41
Cephalothin (USAN)	509	99.80
Cefoxitin (FOX)	47	8.43
**Subclass (penicillins)**		
Ampicillin (AMP)	510	100.00
Amoxicillin-clavulanate (AMC)	456	89.41
Piperacillin-tazobactam (TZP)	138	27.06
**Subclass (carbapenems)**		
Imipenem (IPM)	1	0.12
Meropenem (MEM)	2	0.39
Ertapenem (ETP)	0	0.00
**Subclass (monobactams)**		
Aztreonam (ATM)	506	99.22
**Sulfonamides**		
Trimethoprim-sulfamethoxazole (SXT)	322	63.13
**Aminoglycosides**		
Gentamicin (GEN)	140	27.45
Amikacin (AMK)	2	0.39
**Tetracyclines**		
Tigecycline (TGC)	393	77.05
**Fluoroquinolones**		
Ciprofloxacin (CIP)	347	68.03
Levofloxacin (LVX)	329	64.50
**Nitrofurantoin**		
Nitrofurantoin (NIT)	38	7.45
**Polymyxin**		
Colistin (CST)	298	58.43

**Table 3:** MAR index of ESBL-*E. coli* recovered from urine samples.

Number of Antibiotics	Antibiotic Resistance Profile	No. of Positive Isolates	MAR Index
**15**	CRO, CTX, CAZ, FEP, CXM, USAN, AMP, AMC, ATM, SXT, TGC, CIP, LVX, CST, TZP	163	0.68
**12**	CRO, CTX, CAZ, FEP, CXM, USAN, AMP, AMC, ATM, SXT, GEN, FOX,	139	0.55
**10**	CRO, CTX, CAZ, FEP, CXM, USAN, ATM, SXT, AMP, AMC	121	0.45
**9**	FOX, CTX, ATM, USAN, SXT, CAZ, CTX, TZP, GEN	106	0.41
**8**	CST, CTX, ATM, AMP, AMC, SXT, TGC, CIP	94	0.36
**6**	CXM, AMC, AMP, CRO, CTX, CAZ,	45	0.27
**5**	CRO, FEP, CXM, AMC, LVX	22	0.22
**4**	AMP, CTX, GEN, ATM	18	0.18
**3**	AMP, CXM, SXT	12	0.13

## Data Availability

Not applicable.
